# The CRISPR-Cas Mechanism for Adaptive Immunity and Alternate Bacterial Functions Fuels Diverse Biotechnologies

**DOI:** 10.3389/fcimb.2020.619763

**Published:** 2021-01-28

**Authors:** Sydney Newsom, Hari Priya Parameshwaran, Lindsie Martin, Rakhi Rajan

**Affiliations:** Department of Chemistry and Biochemistry, Price Family Foundation Structural Biology Center, Stephenson Life Sciences Research Center, University of Oklahoma, Norman, OK, United States

**Keywords:** clustered regularly interspaced short palindromic repeats and clustered regularly interspaced short palindromic repeat-associated (CRISPR-Cas), adaptive immunity, Cas9, cascade, gene editing, bacterial pathogenesis, gene regulation, Cas12a

## Abstract

Bacterial and archaeal CRISPR-Cas systems offer adaptive immune protection against foreign mobile genetic elements (MGEs). This function is regulated by sequence specific binding of CRISPR RNA (crRNA) to target DNA/RNA, with an additional requirement of a flanking DNA motif called the protospacer adjacent motif (PAM) in certain CRISPR systems. In this review, we discuss how the same fundamental mechanism of RNA-DNA and/or RNA-RNA complementarity is utilized by bacteria to regulate two distinct functions: to ward off intruding genetic materials and to modulate diverse physiological functions. The best documented examples of alternate functions are bacterial virulence, biofilm formation, adherence, programmed cell death, and quorum sensing. While extensive complementarity between the crRNA and the targeted DNA and/or RNA seems to constitute an efficient phage protection system, partial complementarity seems to be the key for several of the characterized alternate functions. Cas proteins are also involved in sequence-specific and non-specific RNA cleavage and control of transcriptional regulator expression, the mechanisms of which are still elusive. Over the past decade, the mechanisms of RNA-guided targeting and auxiliary functions of several Cas proteins have been transformed into powerful gene editing and biotechnological tools. We provide a synopsis of CRISPR technologies in this review. Even with the abundant mechanistic insights and biotechnology tools that are currently available, the discovery of new and diverse CRISPR types holds promise for future technological innovations, which will pave the way for precision genome medicine.

## Introduction

Clustered Regularly Interspaced Short Palindromic Repeats (CRISPR) and CRISPR- associated (Cas) proteins constitute an RNA-guided adaptive immune system found in several bacteria and most archaea. Over the past decade, detailed molecular mechanisms were discovered which established CRISPR-Cas not just as a phage defense system, but also as a regulator of bacterial physiology such as virulence and group behaviors. (Murugan et al., 2017; Faure et al., 2019; Liu and Doudna, 2020). In this review, we cover the roles of CRISPR-Cas systems within and outside the realm of adaptive immunity and detail the repertoire of biotechnological tools developed from them.

## CRISPR-CAS In Immunity

### Discovery of CRISPR-Cas

CRISPR was discovered over 30 years ago in *Escherichia coli* and later in archaea (Ishino et al., 1987; Mojica et al., 1993). Cas proteins were originally thought to perform DNA repair (Makarova et al., 2002), but were later found to be associated with CRISPR (Jansen et al., 2002). The discovery that spacer sequences match foreign genetic elements led to the hypothesis that CRISPR-Cas is an immune system to protect against invading mobile genetic elements (Bolotin et al., 2005; Mojica et al., 2005; Makarova et al., 2006). Experimental validation of RNA-guided DNA cleavage protecting against intruding phages established CRISPR-Cas as an adaptive immune system (Barrangou et al., 2007).

### Classification of CRISPR-Cas Systems

**Figure 1:**
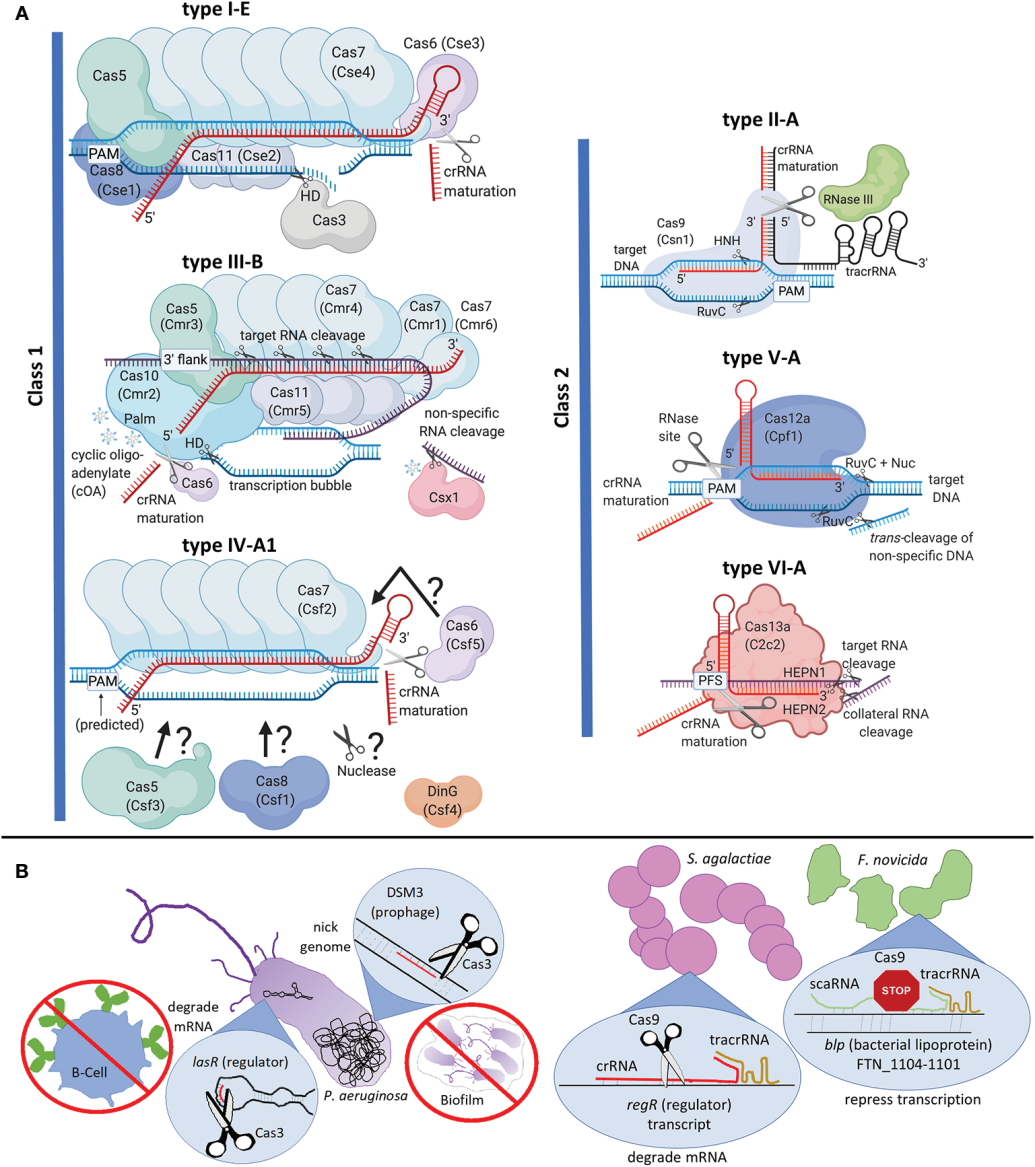
Clustered Regularly Interspaced Short Palindromic Repeats and CRISPR-associated (CRISPR-Cas) classification, interference complexes, and alternate functions. **(A)** Interference complexes, crRNA maturation, and nucleic acid targeting. Type I-E CASCADE from *Escherichia coli* is shown. The interference complex comprises six Cas7s, two Cas11s, and one each of Cas5, Cas8, and Cas6. This complex recruits Cas3, which uses a histidine aspartate (HD) nuclease domain to nick the target DNA, followed by unwinding of the DNA by helicase domain and long-range degradation of DNA by the HD domain. Type III is an RNA-targeting system with a historical distinction into two groups of effector complexes, “Csm” (Cas subtype Mtube, includes subtypes A, D, E and F) and “Cmr” (Cas module RAMP, includes subtypes B and C), differentiated based on the sequences of Cas11 subunit, called as Csm2 and Cmr5 respectively in Csm and Cmr complexes (Tamulaitis et al., 2017). Type III-B from *Pyrococcus furiosus* is shown. The interference complex comprises six Cas7s, coded by three distinct genes (*cmr4*, *cmr1* and *cmr6*), three Cas11s, and one each of Cas5 and Cas10. While Cas7 cleaves target RNA with a 6-nucleotide periodicity, Cas10 cleaves ssDNA in the transcription bubble using its HD domain. The Palm domain of Cas10 produces cyclic oligo-adenylate (cOA) that activates Csx1 for non-specific RNA cleavage. Type IV is represented by subtype A1 from *Aromatoleum aromaticum*. The interference complex comprises Cas5, Cas6, Cas8, and multiple Cas7s, although the exact subunit stoichiometry is unknown. DinG is a helicase necessary for interference. Question marks and arrows in type IV-A1 indicate that subunit organization and nuclease identity is unknown. Cas6, the protein required for crRNA processing may or may not be part of the effector complex in Class I systems. Each class II system is represented by its respective subtype A. Type II-A Cas9 uses its HNH endonuclease domain to cleave the DNA strand hybridized to crRNA and RuvC endonuclease domain to cleave the other strand. RNaseIII is required for crRNA processing in type II systems. Type V-A Cas12a (previously called Cpf1) uses RuvC domain to cleave both strands of the target DNA, even though assistance of the Nuc domain is needed to cleave the strand hybridized to crRNA. Cas12a also possesses non-specific DNA cleavage, called *trans*-cleavage. Type VI-A Cas13a uses two higher eukaryotes and prokaryotes nucleotide-binding (HEPN) domains to elicit sequence-specific and collateral (non-specific) cleavage of target RNA. Type V-A and type VI systems possess specific sites within their effector protein for crRNA processing. Secondary trimming for crRNA maturation is not shown. Protein legacy names are given in parentheses. PAM stands for protospacer adjacent motif, PFS stands for protospacer flanking site, and 3’ flank stands for 3’ flanking sequence of the protospacer that is non-complementary to the 5’-tag of crRNA; each of which are used for self *vs.* non-self recognition. Nucleic acids are not to scale with proteins. Target or non-specifically cleaved RNA is in purple, crRNA is red, tracrRNA is in black and DNA is blue. **(B)** Left: *Pseudomonas aeruginosa* (type I-F) Cas3-mediated RNA and DNA targeting affect host immune evasion and biofilm formation respectively. Right: Comparison between *Streptococcus agalactiae* (type II-A) and *Francisella novicida* (type II-B) mechanisms of Cas9-mediated down-regulation of endogenous genes. The differences include the type of guide RNA (crRNA *vs.* scaRNA), nucleic acid target (mRNA *vs.* genomic DNA), mode of repression (transcript degradation *vs.* steric hindrance to RNA-polymerase), and endogenous gene target (master regulatory protein *vs.* regulon affecting virulence). Created with BioRender.com.

In CRISPR-Cas, the RNA-guided DNA/RNA cleavage occurs through an “effector complex” composed of an RNA guide called CRISPR RNA (crRNA) and a set of Cas proteins (Class 1) or a single multi-domain Cas protein (Class 2) (**Figure 1A**). The two classes are each divided into three types, based on the identity of the signature protein that cleaves the target nucleic acid, and further into several subtypes based on the CRISPR-Cas locus architecture. The most updated classification has 2 classes, 6 types and 33 subtypes (Makarova et al., 2020).

Class 1 CRISPR-Cas systems are most abundant and include types I, III, and IV (**Figure 1A**, Makarova et al., 2015; Makarova et al., 2020). The type I effector complex is called CASCADE (CRISPR-associated complex for antiviral defense), which comprises crRNA and multiple Cas proteins (Brouns et al., 2008; Jore et al., 2011). Once CASCADE locates the target DNA, Cas3 is recruited for DNA cleavage (Sinkunas et al., 2011). The nuclease and helicase activities of Cas3 bring about long-range degradation of intruder DNA (Huo et al., 2014). Type III is unique since it targets and cleaves transcriptionally active RNA using crRNA complementarity, which in turn activates cleavage of the ssDNA associated with the transcription bubble. While Cas7 cleaves the RNA, Cas10 cleaves the ssDNA (Taylor et al., 2015; Mogila et al., 2019; You et al., 2019). Both these activities are essential for full immune protection, with certain systems collaborating with degradosome-nucleases for efficient clearing of intruder nucleic acids (Staals et al., 2013; Staals et al., 2014; Samai et al., 2015; Estrella et al., 2016; Kazlauskiene et al., 2016; Chou-Zheng and Hatoum-Aslan, 2019). Type IV is a minimalistic CRISPR-Cas system devoid of adaptation proteins, mostly found in plasmids or plasmid-like regions and may be involved in plasmid maintenance (Makarova et al., 2020). DinG helicase is essential for plasmid interference in type IV-A, but the nuclease identity is currently unknown (Crowley et al., 2019; Özcan et al., 2019; Pinilla-Redondo et al., 2020).

Class 2 CRISPR-Cas systems consist of types II, V and VI (**Figure 1A**). The type II effector protein Cas9 introduces dsDNA breaks in target DNA using HNH and RuvC endonucleases. Type II systems need an accessory non-coding RNA, *trans*-activating crRNA (tracrRNA), along with crRNA for DNA cleavage (Jinek et al., 2012). Type V systems have Cas12 as the signature effector protein and causes staggered, sequence-specific DNA cleavage using RuvC domain. Subtype-specific variation of the target (DNA *vs.* RNA) and guide RNA requirements [crRNA or crRNA-tracrRNA or crRNA-scout (short-complementarity untranslated)] have also been observed in type V systems (Zetsche et al., 2015, 1; Yan et al., 2019; Harrington et al., 2020). The type VI signature nuclease, Cas13, binds crRNA and locates a complementary RNA target, followed by processive RNase activity using HEPN domains (Abudayyeh et al., 2016; O’Connell, 2019).

### Stages in CRISPR-Cas Defense

There are three distinct stages for CRISPR defense.

(1) Adaptation: During adaptation, a short DNA is excised from intruding DNA and inserted site-specifically into the CRISPR array, creating a new spacer. Known adaptation mechanisms require Cas1 and Cas2 to capture and catalytically insert the spacer. Auxiliary proteins like Cas4, Csn2, Cas9, or non-Cas proteins such as integration host factor are essential for fidelity in certain subtypes. Type III systems with reverse transcriptase-Cas1 fusion proteins can acquire spacers from RNA (Silas et al., 2016). Details of adaptation have been recently reviewed (Sternberg et al., 2016; Mosterd et al., 2020).
(2) crRNA Processing: Typically, the CRISPR array is transcribed into a long pre-crRNA, which associates with Cas proteins for further processing into mature crRNAs. Class 1 systems require Cas6 to process pre-crRNA into individual crRNA molecules (**Figure 1A**, (Staals et al., 2013; Staals et al., 2014; Taylor et al., 2019)). Class 2 systems do not possess a signature Cas protein for crRNA processing, but rather use distinct mechanisms: type II systems depend on RNase III (Deltcheva et al., 2011; Charpentier et al., 2015), type V-A and type VI signature nucleases possess a distinct active site for crRNA processing, and certain type V subtypes depend on host nucleases (East-Seletsky et al., 2016; Fonfara et al., 2016; Liu et al., 2017). Certain type-II systems directly transcribe mature crRNAs using individual promoters within the CRISPR array (Zhang et al., 2013).
(3) Interference: This stage involves the sequence-specific targeting and cleavage of foreign DNA and/or RNA. The classification section above details relevant proteins and cleavage types. Structural and mechanistic details of CRISPR interference have been recently reviewed (Murugan et al., 2017; Liu and Doudna, 2020). Interference involves R-loop formation as the crRNA guide-region hybridizes to target DNA or base-pairing between the crRNA guide region and target RNA. This is followed by cleavage/degradation of the target.

CRISPR-Cas systems must distinguish between self and foreign DNA to avoid self-targeting. The characteristics that warrant intruder cleavage are *(i)* presence of PAM (protospacer adjacent motif), a DNA motif flanking the RNA-DNA complementary region in types I, II, and V; *(ii)* absence of RNA complementarity between the 5′-tag of crRNA and 3′ flank of the target RNA in type III; and *(iii)* presence of a protospacer flanking sequence (PFS), an RNA motif in the target RNA, in type VI (Gleditzsch et al., 2019).

## Physiological Roles of CRISPR-CAS Observed In Bacteria

Some bacteria regulate physiological processes using RNA-guided Cas proteins that target the self-genome. While partial complementarity between the guide RNA and the self-genome or endogenous transcripts seems to drive gene regulation, complete complementarity with the genome has been shown to regulate prophage and temperate phage life-cycles and trigger bacterial evolution through recombination to avoid lethal self-targeting (Aklujkar and Lovley, 2010; Vercoe et al., 2013; Goldberg et al., 2014). We review here the roles of CRISPR systems in bacterial pathogenicity and survival.

### Virulence

#### Host Immune Evasion

CRISPR-Cas can regulate gene expression to conceal bacteria from host toll-like receptors (TLR). The best studied example is the *Francisella novicida* U112 type II-B system, which downregulates a bacterial lipoprotein (BLP) on the cell envelope to evade host immune responses (Sampson et al., 2013; Ratner et al., 2019). This mechanism requires the association of Cas9 with tracrRNA and small, CRISPR-Cas-associated RNA (scaRNA), which specifically represses transcription of a regulon containing *blp* (FTN_1104-1101) (**Figure 1B**). Transcriptomic studies have revealed similar trends where *blp*s are upregulated in *cas9* deletion mutants (Δ*cas9*) of *Streptococcus agalactiae* GD201008-001 (type II-A) and *Riemerella anatipestifer* (type II-C) (Ma et al., 2018; Wang et al., 2019). In *Streptococcus pyogenes* GAS-M1T1-5448 (type II-A), Δ*cas9* produces less master regulator protein Mga, which in turn downregulates ScpA and SIC proteins, which are essential to inactivate the host complement immune defense (Gao et al., 2019). *S. pyogenes* Cas9 also downregulates the CovR/S two component system (TCS) which regulates capsule genes that confer antiphagocytic properties (Sarkar and Sumby, 2017; Gao et al., 2019). Involvement of a type I-F CRISPR-system in virulence was demonstrated in *Pseudomonas aeruginosa* UCBPP-PA14, where Cas3 degrades the mRNA of the master regulator protein LasR to evade the TLR-initiated host immune response (**Figure 1B**, (Li et al., 2016)). The bacterial molecule regulated to avoid recognition is currently unknown.

#### Bacterial Adherence and Cytotoxicity

CRISPR-Cas systems modulate bacterial-host interactions, the mechanisms of which are currently evolving. Relative to the wild-type, *Neisseria*, *Streptococcus*, and *Campylobacter* △*cas9* strains adhere poorly to mammalian host cells (Ma et al., 2018; Shabbir et al., 2018; Heidrich et al., 2019; Spencer et al., 2019; Gao et al., 2019). The *S. pyogenes* mechanism involves Cas9-mediated down-regulation of the FasA/FasB TCS that downregulates adhesion factors (Gao et al., 2019). *Campylobacter jejuni* and *S. agalactiae* Cas9s can increase bacterial cytotoxicity, adhesion, and survival within hosts cells (Louwen et al., 2013; Ma et al., 2018; Shabbir et al., 2018; Saha et al., 2020a). In *S. agalactiae*, hyaluronidase activity essential for blood-brain barrier penetration is regulated *via* Cas9-mediated cleavage of the master regulator *regR* transcript (**Figure 1B**, (Ma et al., 2018)).

#### Group Behavior

There is growing evidence that CRISPR systems can regulate group behaviors to increase bacterial virulence. Based on *Salmonella enterica* serovar Enteritidis △*cas3* (type I-E) transcriptome studies, Cas3 downregulates LsrF production, preventing degradation of quorum sensing (QS) signaling molecules by LsrF, ultimately increasing expression of the *lsr* QS operon (Cui et al., 2020). Transcriptomic studies of △*cas3 Streptococcus mutans* UA159 (type I-C) showed downregulation of biofilm formation genes controlled by the VicR/K TCS, which is known to influence streptococcal tissue specificity (Vega et al., 2016; Tang et al., 2019). Phage-dependent biofilm regulation was observed in *Pseudomonas aeruginosa* strain UCBPP-PA14, where partial crRNA hybridization to lysogenized DMS3 phage recruits Cas3 (type I-F) to nick the bacterial genome, initiating a RecA-dependent SOS response that inhibits biofilm formation and swarming (**Figure 1B**, (Heussler et al., 2015)). Experiments have implicated Cas9-based virulent group behaviors in *C. jejuni* (type II-C) (Shabbir et al., 2018), *S. pyogenes* (type II-A) (Gao et al., 2019), *Neisseria meningitidis* (type II-C) (Sampson et al., 2013), and *S. agalactiae* (type II-A) (Ma et al., 2018) where △*cas9* strains had decreased biofilm formation.

### Relation to Genome Repair and Involvement in Non-Virulent Gene Regulation and Programmed Cell Death

Current research also shows CRISPR-Cas systems regulating genes not directly involved in bacterial virulence. Experimental evidence has demonstrated Cas1’s ability to cleave branched DNA substrate and revealed enhanced sensitivity of △*cas1 E. coli* to DNA damage (Babu et al., 2011). A recent study has shown that non-homologous end-joining (NHEJ) does not co-exist with type II-A systems due to the competition between Csn2 (type II-A) and NHEJ proteins for DNA substrates. Another DNA-repair system, RecBCD, is essential for spacer acquisition in *E. coli* (type I-E) (Levy et al., 2015). These studies suggest complex interdependence of CRISPR and DNA repair systems, the mechanisms of which are still elusive. Transcriptomic and proteomic studies of △*cas9 S. pyogenes* (type II-A) (Gao et al., 2019), Group B *Streptococcus* (type II-A) (Spencer et al., 2019), and *Riemerella anatipestifer* (type II-C) (Wang et al., 2019) have indicated both up- and down- regulation of several endogenous genes, the implications of which are not clear. Several studies in *Myxococcus xanthus* have shown that CRISPR systems are involved in cell-stress dependent sporulation (type I-C) and fruiting body development (type III-B) (Viswanathan et al., 2007; Wallace et al., 2014).

Type III and type VI CRISPR systems promote indiscriminate RNA cleavage leading to PCD, a strategy employed when immune protection has failed (Faure et al., 2019). In type III systems, crRNA binding to target RNA activates production of cyclic oligoadenylate (cOA) by Cas10. The cOA molecules initiate Csm6/Csx1’s indiscriminate RNase activity resulting in cell death (Kazlauskiene et al., 2017; Niewoehner et al., 2017). In type VI, crRNA binding to target RNA activates Cas13 for promiscuous RNase activity (Abudayyeh et al., 2016). Interestingly, the resemblance of Cas2 to the toxin component of toxin-antitoxin systems and the genomic co-localization of CRISPR and PCD elements have led to the idea of their co-dependence in bacterial physiology, and are awaiting experimental conformation (reviewed in Faure et al., 2019).

### Diversity of CRISPR-Cas Mechanisms

Current studies indicate that CRISPR has functions beyond adaptive immunity, primarily in regulating genome content and gene expression. Horizontal Gene Transfer (HGT) can be negatively impacted in CRISPR-containing bacteria since the acquired DNA can be targeted by CRISPR. The effects of CRISPR on HGT are evolving. Some bacteria compensate limitations on HGT by maintaining a defective CRISPR locus or establishing mechanisms for CRISPR-tolerance (Sampson and Weiss, 2013; Wimmer and Beisel, 2020). A different perspective on offsetting this disadvantage has been demonstrated by CRISPR-mediated enhancement of transduction by phages, which then enables transfer of genetic materials between bacteria (Watson et al., 2018).

Based on studies of streptococcal physiology, we speculate that CRISPR-mediated gene regulation promotes strain differentiation *via* genome remodeling and regulatory changes. CRISPR changes expression of *S. pyogenes* master regulators Mga and VicR and the TCS CovR/S, which then regulate immunomodulatory virulence factors that drive development of strains with different host tissue preferences and physiologies ranging from hypervirulent to carrier status (Vega et al., 2016; Sarkar and Sumby, 2017). Similarly, natural mutation in *P. aeruginosa* master regulator LasR increases bacterial fitness in the cystic fibrosis lung (Smith et al., 2006). A common theme in these diverse mechanisms is that Cas proteins target genes that bring about differential phenotypes in bacteria. *Legionella pneumophila*’s requirement of Cas2 to infect amoebas indicates the involvement of other Cas proteins in endogenous gene regulation, requiring future studies to unravel more of such mechanisms.

## CRISPR-CAS Biotechnology Tools

The RNA-guided nucleic acid targeting of Cas proteins offer several biotechnology tools dependent on the diverse CRISPR mechanisms (**Table 1**).We are providing selected aspects of currently available applications (**Table 1**), with more details in **Table S1** and several recent reviews (Pickar-Oliver and Gersbach, 2019; Xu and Qi, 2019).

**Table 1:** Clustered Regularly Interspaced Short Palindromic Repeats and CRISPR-associated (CRISPR-Cas) biotechnology applications.

Biotechnology Application	CRISPR-Cas system and relevant activities	References
A. Gene editing	1. SpyCas9 (type II-A) and its high-fidelity variants; light and chemically induced Cas9 expression; sgRNA regulation to control Cas9 2. Cas12a (type V-A), sequence specific DNA cleavage producing staggered ends 3. CASCADE and Cas3 for sequence-specific DNA degradation and Cas8-FokI fusion for sequence-specific DNA cleavage 4. Cmr (type III-B), sequence specific interference of transcriptionally active DNA	1. (Jinek et al., 2012; Cho et al., 2013; Nihongaki et al., 2015; Kleinstiver et al., 2016; Slaymaker et al., 2016; Chen et al., 2017; Maji et al., 2017) 2. (Hur et al., 2016; Yan et al., 2017) 3. (Cameron et al., 2019) 4. (Li et al., 2015)
B. Base editing	SpyCas9 fused to cytidine deaminase or adenosine to inosine deaminase; Cas12a fused to cytidine deaminase; Cas13 fused to adenosine deaminase	(Komor et al., 2016; Cox et al., 2017; Gaudelli et al., 2017; Li et al., 2018)
C. Gene knockdown *via* ssRNA cleavage	Cas9, Cmr, Cas13-based methods	(Zebec et al., 2014; Abudayyeh et al., 2017; Batra et al., 2017; Konermann et al., 2018; Strutt et al., 2018)
D. Transcriptional repression	Nuclease inactivated SpyCas9 and its tag-fusion derivatives, nuclease inactivated Cas12a, CASCADE without Cas3	(Qi et al., 2013; Rath et al., 2014; Huang et al., 2017; Zhang et al., 2017)
E. Gene activation	Fusions of SpyCas9, Cas12a, and CASCADE to transcriptional activation domains	(Perez-Pinera et al., 2013; Breinig et al., 2019; Young et al., 2019)
F. Nucleic acid detection	Cas12a, Cas13, Csm6	(Gootenberg et al., 2018)

### Gene Editing

The revolutionary characteristic of CRISPR-Cas-based gene editing is the ability to introduce heritable genome modifications by programing Cas proteins using guide RNAs, instead of modifying the editing protein, which was the limiting aspect of zinc fingers and TALENS (Gaj et al., 2013).

The most widely used CRISPR-based gene editing system is *S. pyogenes* Cas9 (SpyCas9) with a single guide RNA (sgRNA) (**Table 1**.A). The most prominent application is modification of eukaryotic genes utilizing efficient DNA-repair pathways of eukaryotes to repair Cas9 induced DNA breakage by NHEJ (creates gene knock-outs) or homology directed repair (HDR, creates gene knock-ins). An approach to fix genetic mutations while eliminating unwarranted effects from off-target DNA cleavage is fusing Cas9 with base editors (**Table 1**.B, (Komor et al., 2016)). Cas9-based gene editing in prokaryotes is limited due to inefficient DNA repair mechanisms; however, Cas9 cleavage-induced killing of unedited cells can be used to increase efficiency of other bacterial gene-editing methods (Wilson et al., 2003).

Cas12a and CASCADE gene editing systems complement those of Cas9 since their DNA cleavage mechanisms produce HDR-enhancing staggered ends and instill long-range deletions respectively (Dolan et al., 2019; Paul and Montoya, 2020).

### Transcript and Epigenetic Manipulation

Transcripts can be targeted and cleaved by Cas13 and some Cas9 orthologs including *Staphylococcus aureus* Cas9 for transcript level gene knock-down (**Table 1**.C, (Konermann et al., 2018; Strutt et al., 2018)). Nuclease inactivated dead Cas (dCas) proteins can block or activate transcription *via* steric hindrance of RNA-polymerase or fusion to transcriptional activators respectively (**Table 1**.D and E, (Perez-Pinera et al., 2013; Qi et al., 2013)).

### Nucleic Acid Detection Kits

Cas12a and Cas13 inherently cleave non-specific nucleic acids after RNA-mediated target binding (**Figure 1A**), enabling several fluorescent-based detection kits (**Table 1**.F, (Gootenberg et al., 2018)). Notably, STOP (SHERLOCK Testing in One Pot) for COVID-19 diagnostics (STOPCovid) uses this technology (Joung et al., 2020).

### Biomedical Tools and Future Prospects in Genome Medicine

The site-specific genetic modulation facilitated by Cas proteins has been monumental in quickly advancing gene therapy with several ongoing clinical trials for cancer immunotherapies, blood disorders, blindness *etc*. (**Table S1**.A.6, clinicaltrials.gov). To facilitate progress, SpyCas9 off-targeting concerns are being addressed using highly stringent Cas proteins, temporal control of protein expression, and gene editing reaction activators/quenchers (**Table S1**.A, (Chen et al., 2017)). As new CRISPR systems with diverse mechanisms are being characterized, new biomedical applications will follow.

## Outlook

As described here, CRISPR-Cas is a two-in-one mechanism for protection against intruding nucleic acids as well as for regulating bacterial physiology, including pathogenicity. Following the current discovery trends, genomic analyses will keep unearthing new CRISPR-Cas systems, sometimes even re-writing the existing rules (e.g. discovery of first known Cas9 in nanoarchaea, (Burstein et al., 2017)). The mechanistic basis of CRISPR-based physiology is still evolving. Interestingly, the majority of spacer targets are yet to be identified, (Shmakov et al., 2017) and these target locations may hold the key to mechanisms of more CRISPR-mediated alternate functions.

Currently, Cas9, Cas1, Cas2, and Cas3 have been directly implicated in several physiological functions, suggesting potential comparable functions in other Cas proteins. So far, Cas9 has been the most divergent in function ranging from phage defense to gene regulation to directly acting as a virulence factor in *C. jejuni* (Faure et al., 2019). Future research is crucial in understanding the structure-function relations of these diverse Cas9 mechanisms. The recent finding that secretion of guide-free Cas9 by *C. jejuni* can inflict host cell DNA damage points to Cas9 mechanisms that are independent of guide RNA (Sundaresan et al., 2017; Saha et al., 2020b).

The arms race between bacteria and viruses has delivered an arsenal of anti-CRISPR proteins (Marino et al., 2020). In an interesting twist, the use of CasΦ by huge phages showed adaptation of CRISPR-Cas mechanisms to ward off virophages (Pausch et al., 2020). The fitness cost benefits of an active CRISPR system and the maintenance of degenerated CRISPR-Cas systems in several bacteria provide interesting future research avenues. CRISPR-Cas systems have proven unique because of their repurposing into powerful diagnostic, therapeutical and experimental tools. While there are several aspects such as off-target effects and promiscuous DNA damage that need to be fixed by future research (Haapaniemi et al., 2018; Wang et al., 2020), the days of personal precision genome medicine are a step closer with this powerful technology.

## Author Contributions

SN, HPP, LM, and RR wrote, reviewed, and edited the manuscript. All authors contributed to the article and approved the submitted version.

## Funding

The preparation of this review article was supported by a National Science Foundation grant (grant number MCB-1716423) and by a grant from the Research Council of the University of Oklahoma Norman Campus to RR.

## Conflict of Interest

The authors declare that the research was conducted in the absence of any commercial or financial relationships that could be construed as a potential conflict of interest.

## Abbreviations

BLP, bacterial lipoprotein; Cas, CRISPR-associated; cOA, cyclic oligo-adenylate; CovR/S, control of virulence regulator and sensor; CRISPR, clustered, regularly interspaced short palindromic repeats; crRNA, CRISPR RNA; dCas, nuclease inactivated dead Cas protein; ds, double stranded; FasA/FasB, fibronectin/fibrinogen-binding/hemolytic activity/streptokinase regulatory (Fas) system; HD, histidine and aspartate; HDR, homology directed repair; HEPN, higher eukaryotes and prokaryotes nucleotide-binding; HGT, horizontal gene transfer; LasR, acyl-homoserine-lactone quorum sensing circuit regulator; LsrF, 3-hydroxy-5-phosphonooxypentane-2,4-dione thiolase; Mga, multigene regulator in GAS; NHEJ, non-homologous end joining; PCD, programmed cell death; QS, quorum sensing; scaRNA, small, CRISPR-Cas-associated RNA; ScpA, streptococcal C5a peptidase; sgRNA, single guide RNA; SIC, streptococcal inhibitor of complement; SpyCas9, *Streptococcus pyogenes* Cas9; ss, single stranded; TALENS, Transcription activator-like effector nucleases; TCS, two-component system; TLR, toll-like receptor; tracrRNA, *trans*-activating crRNA; VicR/K, a TCS with a putative histidine kinase (VicK) and a response regulator (VicR).
