# Supplementary material for: The CRISPR-Cas Mechanism for Adaptive Immunity and Alternate Bacterial Functions Fuels Diverse Biotechnologies
Source: Front Cell Infect Microbiol. 2021 Jan 28;10:619763. doi: 10.3389/fcimb.2020.619763 (PMC7876343; doi:10.3389/fcimb.2020.619763)
Supplement: Supplementary file 1 [file Table_1.docx]

**The CRISPR-Cas mechanism for adaptive immunity and alternate bacterial functions fuels diverse biotechnologies**

#

**Sydney Newsom^1#^, Hari Priya Parameshwaran^1#^, Lindsie Martin^1#^, Rakhi Rajan^1*^**

^#^These authors contributed equally.

^1^Department of Chemistry and Biochemistry, Price Family Foundation Structural Biology Center, Stephenson Life Sciences Research Center, University of Oklahoma, Norman, OK, USA

*****To whom correspondence should be addressed. Email: [r-rajan@ou.edu](mailto:r-rajan@ou.edu)

Supplementary Table 1

**Supplementary Table 1. Expanded CRISPR-Cas biotechnology applications**

| **Biotechnology Application** | **Cas Protein and CRISPR-Cas type, relevant activities, modifications** | **References** |
| --- | --- | --- |
| A. Gene editing | 1a. SpyCas9 (type II-A), sequence specific DNA cleavage producing a mixture of staggered and blunt ends  1b. Hyper-accurate Cas9 (HypaCas9), four amino acid mutations near the RNA-DNA interface  1c. High-fidelity SpyCas9 (SpCas9-HF), mutation of four amino acids that non-specifically contact target DNA  1d. Enhanced specificity SpyCas9 (eSpCas9), three amino acid mutations in a groove that stabilizes the non-target strand of the target DNA  1e. Light activated Cas9, split into two fragments and fused to dimerization domains  1f. sgRNA regulation to control Cas9  1g. chemically inducible Cas9, various modifications  2. Cas12a (type V-A), sequence specific DNA cleavage producing staggered ends  3. CASCADE (type 1-E), sequence specific DNA binding, fused to FokI nuclease  4. CASCADE and Cas3 (type I-A), sequence specific DNA cleavage and degradation of one of the strands of a DNA  5. Cmr (type III-B), sequence specific interference of transcriptionally active DNA  6. Direct injection of CRISPR-Cas-based drug into the retina to treat hereditary blindness  7. Cas10-Csm and Csm6 (type III-A), viral DNA and RNA cleavage that increases viral gene editing efficiency by selecting against unedited phage  8. Anti-CRISPR proteins, CRISPR-Cas inhibition as a selectable marker for editing viral genomes  9. Anti-CRISPR proteins, CRISPR-Cas inhibition quenches gene editing reactions to minimize off-targeting | 1a. (Jinek et al., 2012; Cho et al., 2013; Jiang et al., 2013; Chu et al., 2015)  1b. (Chen et al., 2017)  1c. (Kleinstiver et al., 2016)  1d. (Slaymaker et al., 2016)  1e. (Nihongaki et al., 2015; Yu et al., 2020)  1f. (Lee et al., 2016; Liu et al., 2016b; Ferry et al., 2017; Maji et al., 2017; Tang et al., 2017)  1g. (Davis et al., 2015; Dow et al., 2015; Zetsche et al., 2015; Liu et al., 2016a; Maji et al., 2017)  2. (Hur et al., 2016; Ferenczi et al., 2017; Yan et al., 2017)  3. (Cameron et al., 2019)  4 & 5. (Li et al., 2015)  6. ClinicalTrials.gov Identifier: NCT03872479  7. (Nayeemul Bari and Hatoum-Aslan, 2019)  8. (Mayo-Muñoz et al., 2018)  9. (Marino et al., 2020) |
| B. Base editing | 1. SpyCas9 nickase (type II-A), sequence specific DNA binding, fused to both cytidine deaminase and uracil DNA glycosylase inhibitor  2. SpyCas9 (type II-A), sequence specific DNA binding, nuclease inactivated and fused to an engineered adenosine to inosine deaminase capable of targeting DNA  3. nuclease inactivated Cas12a (type V-A), sequence specific DNA binding, fused to cytidine deaminase and uracil DNA glycosylase inhibitor  4. nuclease inactivated Cas13 (type VI-B), sequence specific RNA binding, fused to adenosine deaminase | 1. (Komor et al., 2016)  2. (Gaudelli et al., 2017)  3. (Li et al., 2018)  4. (Cox et al., 2017) |
| C. Gene knockdown via ssRNA cleavage | 1. *Staphylococcus aureus* Cas9 (II-A), *Neisseria meningitidis* Cas9 (II-C) ssRNA cleavage  2. Cmr (III-B), ssRNA cleavage  3. Cas13 (VI-A and D), crRNA maturation and ssRNA cleavage  4. nuclease inactivated SpyCas9 (type II-A) fused to RNA endonuclease domain, sequence specific ssRNA binding | 1. (Rousseau et al., 2018; Strutt et al., 2018)  2. (Zebec et al., 2014)  3. (Abudayyeh et al., 2017; Konermann et al., 2018)  4. (Batra et al., 2017) |
| D. Transcriptional repression | 1. nuclease inactivated SpyCas9 (type II-A), sequence specific DNA binding  2. nuclease inactivated SpyCas9 (type II-A) fused to an epitope tag that recruits antibody-fused methyltransferase that methylates the genomic target to repress transcription, sequence specific DNA binding  3. nuclease inactivated Cas12a (type V-A), crRNA maturation and sequence specific DNA binding  4. CASCADE (type I-E), DNA binding | 1. (Qi et al., 2013)  2. (Huang et al., 2017)  3. (Zhang et al., 2017)  4. (Rath et al., 2014) |
| E. Gene activation | 1a. nuclease inactivated SpyCas9 (type II-A) fused to a transactivation domain, sequence specific DNA binding  1b. Light activated SpyCas9, nuclease inactivated and fused to a light-inducible heterodimerizing protein  2. Cas12a (type V-A), crRNA maturation and sequence specific DNA binding, fused to a transactivation domain  3. CASCADE (type I-E), crRNA maturation and sequence specific DNA binding, fused to plant transcriptional activation domain | 1a. (Perez-Pinera et al., 2013)  1b. (Polstein and Gersbach, 2015)  2. (Breinig et al., 2019)  3. (Young et al., 2019) |
| F. Nucleic acid detection | 1. Cas12a (type V-A), collateral ssDNA cleavage activity upon target binding  2. Cas13 (type VI-A and B), collateral ssRNA cleavage activity upon target binding  3. Csm6 (type III-A), non-specific nuclease | 1-3. (Gootenberg et al., 2018)  1. (Chen et al., 2018) |
| G. Antimicrobial | 1. SpyCas9 (type II-A), dsDNA interference  2. CASCADE and Cas3 (type I-E), dsDNA interference  3. Cas13a (type VI-A), promiscuous ssRNA cleavage  4. Anti-CRISPR proteins, CRISPR-Cas inhibition to expand the host range for phage-derived therapeutics | 1. (Citorik et al., 2014)  2. (Gomaa et al., 2014)  3. (Kiga et al., 2020)  4. (Marino et al., 2020) |
| H. Transposition or genome tagging | 1. CASCADE (type I-F), sequence specific DNA binding, fused to transposition protein  2. Cas1 and Cas2 (type I-E), sequence-selective DNA integration | 1. (Klompe et al., 2019)  2. (Wright et al., 2017) |
| I. Protect plants from both RNA and DNA viruses | 1. Cas13 (type VI-A), crRNA maturation and sequence specific ssRNA cleavage  2. SpyCas9 (type II-A), sequence specific DNA interference | 1. (Aman et al., 2018)  2. (Ali et al., 2015) |
| J. Decrease bacterial pathogenicity | Anti-CRISPR proteins to inhibit CRISPR-Cas systems involved in bacterial pathogenicity | (Marino et al., 2020) |
| K. Record transcription | Cas1:Cas2 complex (type III-D), natural fusion to reverse transcriptase, reverse transcription and sequence selective DNA integration | (Schmidt et al., 2018) |
| L. Store digital information | Cas1:Cas2 complex (type I-E), sequence selective DNA integration | (Shipman et al., 2017) |
| M. Visualizing nucleic acid localization | 1. nuclease inactivated SpyCas9 (type II-A) fused to GFP, sequence specific binding to target DNA  2. nuclease inactivated Cas13 (type VI-A) fused to GFP, sequence specific ssRNA binding | 1. (Chen et al., 2013; Nelles et al., 2016)  2. (Abudayyeh et al., 2017) |
| N. Mediate chromatin immunoprecipitation for downstream analysis of epiproteome (interacting proteins, histone post translational modifications, RNAs, and neighboring genomic regions) via mass spectrometry and nucleic acid sequencing | nuclease inactivated and affinity tagged SpyCas9 (type II-A), sequence specific DNA binding | (Waldrip et al., 2014; Fujita and Fujii, 2015) |
| O. Subnuclear proteomic profiling | nuclease inactivated SpyCas9 (type II-A) fused to APEX2 (an engineered soybean ascorbate peroxidase that labels near-by proteins with biotin), sequence specific DNA binding | (Gao et al., 2019) |
| P. Influence mRNA splicing outcomes without hindering translation to protein (potential treatment for diseases caused by gene mis-splicing) | Cas13d (CasRx, type VI-D), sequence specific DNA binding, nuclease inactivated | (Konermann et al., 2018) |

**Reference List**

Abudayyeh, O. O., Gootenberg, J. S., Essletzbichler, P., Han, S., Joung, J., Belanto, J. J., et al. (2017). RNA targeting with CRISPR–Cas13. *Nature* 550, 280–284. doi:10.1038/nature24049.

Ali, Z., Abulfaraj, A., Idris, A., Ali, S., Tashkandi, M., and Mahfouz, M. M. (2015). CRISPR/Cas9-mediated viral interference in plants. *Genome Biology* 16, 238. doi:10.1186/s13059-015-0799-6.

Aman, R., Ali, Z., Butt, H., Mahas, A., Aljedaani, F., Khan, M. Z., et al. (2018). RNA virus interference via CRISPR/Cas13a system in plants. *Genome Biology* 19, 1. doi:10.1186/s13059-017-1381-1.

Batra, R., Nelles, D. A., Pirie, E., Blue, S. M., Marina, R. J., Wang, H., et al. (2017). Elimination of Toxic Microsatellite Repeat Expansion RNA by RNA-Targeting Cas9. *Cell* 170, 899-912.e10. doi:10.1016/j.cell.2017.07.010.

Breinig, M., Schweitzer, A. Y., Herianto, A. M., Revia, S., Schaefer, L., Wendler, L., et al. (2019). Multiplexed orthogonal genome editing and transcriptional activation by Cas12a. *Nature Methods* 16, 51–54. doi:10.1038/s41592-018-0262-1.

Cameron, P., Coons, M. M., Klompe, S. E., Lied, A. M., Smith, S. C., Vidal, B., et al. (2019). Harnessing type I CRISPR–Cas systems for genome engineering in human cells. *Nature Biotechnology* 37, 1471–1477. doi:10.1038/s41587-019-0310-0.

Chen, B., Gilbert, L. A., Cimini, B. A., Schnitzbauer, J., Zhang, W., Li, G.-W., et al. (2013). Dynamic Imaging of Genomic Loci in Living Human Cells by an Optimized CRISPR/Cas System. *Cell* 155, 1479–1491. doi:10.1016/j.cell.2013.12.001.

Chen, J. S., Dagdas, Y. S., Kleinstiver, B. P., Welch, M. M., Sousa, A. A., Harrington, L. B., et al. (2017). Enhanced proofreading governs CRISPR–Cas9 targeting accuracy. *Nature* 550, 407–410. doi:10.1038/nature24268.

Chen, J. S., Ma, E., Harrington, L. B., Da Costa, M., Tian, X., Palefsky, J. M., et al. (2018). CRISPR-Cas12a target binding unleashes indiscriminate single-stranded DNase activity. *Science* 360, 436. doi:10.1126/science.aar6245.

Cho, S. W., Kim, S., Kim, J. M., and Kim, J.-S. (2013). Targeted genome engineering in human cells with the Cas9 RNA-guided endonuclease. *Nature Biotechnology* 31, 230–232. doi:10.1038/nbt.2507.

Chu, V. T., Weber, T., Wefers, B., Wurst, W., Sander, S., Rajewsky, K., et al. (2015). Increasing the efficiency of homology-directed repair for CRISPR-Cas9-induced precise gene editing in mammalian cells. *Nature Biotechnology* 33, 543–548. doi:10.1038/nbt.3198.

Citorik, R. J., Mimee, M., and Lu, T. K. (2014). Sequence-specific antimicrobials using efficiently delivered RNA-guided nucleases. *Nature Biotechnology* 32, 1141–1145. doi:10.1038/nbt.3011.

Cox, D. B. T., Gootenberg, J. S., Abudayyeh, O. O., Franklin, B., Kellner, M. J., Joung, J., et al. (2017). RNA editing with CRISPR-Cas13. *Science* 358, 1019–1027. doi:10.1126/science.aaq0180.

Davis, K. M., Pattanayak, V., Thompson, D. B., Zuris, J. A., and Liu, D. R. (2015). Small molecule–triggered Cas9 protein with improved genome-editing specificity. *Nature Chemical Biology* 11, 316–318. doi:10.1038/nchembio.1793.

Dow, L. E., Fisher, J., O’Rourke, K. P., Muley, A., Kastenhuber, E. R., Livshits, G., et al. (2015). Inducible *in vivo* genome editing with CRISPR-Cas9. *Nature Biotechnology* 33, 390–394. doi:10.1038/nbt.3155.

Ferenczi, A., Pyott, D. E., Xipnitou, A., and Molnar, A. (2017). Efficient targeted DNA editing and replacement in *Chlamydomonas reinhardtii* using Cpf1 ribonucleoproteins and single-stranded DNA. *Proc Natl Acad Sci USA* 114, 13567. doi:10.1073/pnas.1710597114.

Ferry, Q. R. V., Lyutova, R., and Fulga, T. A. (2017). Rational design of inducible CRISPR guide RNAs for *de novo* assembly of transcriptional programs. *Nature Communications* 8, 14633. doi:10.1038/ncomms14633.

Fujita, T., and Fujii, H. (2015). “Isolation of Specific Genomic Regions and Identification of Associated Molecules by Engineered DNA-Binding Molecule-Mediated Chromatin Immunoprecipitation (enChIP) Using CRISPR,” in *Chromatin Protocols*, ed. S. P. Chellappan (New York, NY: Springer New York), 43–52. doi:10.1007/978-1-4939-2474-5_4.

Gao, X. D., Rodríguez, T. C., and Sontheimer, E. J. (2019). “Chapter Sixteen - Adapting dCas9-APEX2 for subnuclear proteomic profiling,” in *Methods in Enzymology*, ed. S. Bailey (Academic Press), 365–383. doi:10.1016/bs.mie.2018.10.030.

Gaudelli, N. M., Komor, A. C., Rees, H. A., Packer, M. S., Badran, A. H., Bryson, D. I., et al. (2017). Programmable base editing of A•T to G•C in genomic DNA without DNA cleavage. *Nature* 551, 464–471. doi:10.1038/nature24644.

Gomaa, A. A., Klumpe, H. E., Luo, M. L., Selle, K., Barrangou, R., and Beisel, C. L. (2014). Programmable removal of bacterial strains by use of genome-targeting CRISPR-Cas systems. *mBio* 5, e00928. doi:10.1128/mBio.00928-13.

Gootenberg, J. S., Abudayyeh, O. O., Kellner, M. J., Joung, J., Collins, J. J., and Zhang, F. (2018). Multiplexed and portable nucleic acid detection platform with Cas13, Cas12a, and Csm6. *Science* 360, 439. doi:10.1126/science.aaq0179.

Huang, Y.-H., Su, J., Lei, Y., Brunetti, L., Gundry, M. C., Zhang, X., et al. (2017). DNA epigenome editing using CRISPR-Cas SunTag-directed DNMT3A. *Genome Biology* 18, 176. doi:10.1186/s13059-017-1306-z.

Hur, J. K., Kim, K., Been, K. W., Baek, G., Ye, S., Hur, J. W., et al. (2016). Targeted mutagenesis in mice by electroporation of Cpf1 ribonucleoproteins. *Nature Biotechnology* 34, 807–808. doi:10.1038/nbt.3596.

Jiang, W., Bikard, D., Cox, D., Zhang, F., and Marraffini, L. A. (2013). RNA-guided editing of bacterial genomes using CRISPR-Cas systems. *Nature Biotechnology* 31, 233–239. doi:10.1038/nbt.2508.

Jinek, M., Chylinski, K., Fonfara, I., Hauer, M., Doudna, J. A., and Charpentier, E. (2012). A Programmable Dual-RNA–Guided DNA Endonuclease in Adaptive Bacterial Immunity. *Science* 337, 816. doi:10.1126/science.1225829.

Kiga, K., Tan, X.-E., Ibarra-Chávez, R., Watanabe, S., Aiba, Y., Sato’o, Y., et al. (2020). Development of CRISPR-Cas13a-based antimicrobials capable of sequence-specific killing of target bacteria. *Nature Communications* 11, 2934. doi:10.1038/s41467-020-16731-6.

Kleinstiver, B. P., Pattanayak, V., Prew, M. S., Tsai, S. Q., Nguyen, N. T., Zheng, Z., et al. (2016). High-fidelity CRISPR–Cas9 nucleases with no detectable genome-wide off-target effects. *Nature* 529, 490–495. doi:10.1038/nature16526.

Klompe, S. E., Vo, P. L. H., Halpin-Healy, T. S., and Sternberg, S. H. (2019). Transposon-encoded CRISPR–Cas systems direct RNA-guided DNA integration. *Nature* 571, 219–225. doi:10.1038/s41586-019-1323-z.

Komor, A. C., Kim, Y. B., Packer, M. S., Zuris, J. A., and Liu, D. R. (2016). Programmable editing of a target base in genomic DNA without double-stranded DNA cleavage. *Nature* 533, 420–424. doi:10.1038/nature17946.

Konermann, S., Lotfy, P., Brideau, N. J., Oki, J., Shokhirev, M. N., and Hsu, P. D. (2018). Transcriptome Engineering with RNA-Targeting Type VI-D CRISPR Effectors. *Cell* 173, 665-676.e14. doi:10.1016/j.cell.2018.02.033.

Lee, Y. J., Hoynes-O’Connor, A., Leong, M. C., and Moon, T. S. (2016). Programmable control of bacterial gene expression with the combined CRISPR and antisense RNA system. *Nucleic Acids Research* 44, 2462–2473. doi:10.1093/nar/gkw056.

Li, X., Wang, Y., Liu, Y., Yang, B., Wang, X., Wei, J., et al. (2018). Base editing with a Cpf1–cytidine deaminase fusion. *Nature Biotechnology* 36, 324–327. doi:10.1038/nbt.4102.

Li, Y., Pan, S., Zhang, Y., Ren, M., Feng, M., Peng, N., et al. (2015). Harnessing Type I and Type III CRISPR-Cas systems for genome editing. *Nucleic Acids Research* 44, e34–e34. doi:10.1093/nar/gkv1044.

Liu, K. I., Ramli, M. N. B., Woo, C. W. A., Wang, Y., Zhao, T., Zhang, X., et al. (2016a). A chemical-inducible CRISPR–Cas9 system for rapid control of genome editing. *Nature Chemical Biology* 12, 980–987. doi:10.1038/nchembio.2179.

Liu, Y., Zhan, Y., Chen, Z., He, A., Li, J., Wu, H., et al. (2016b). Directing cellular information flow via CRISPR signal conductors. *Nature Methods* 13, 938–944. doi:10.1038/nmeth.3994.

Maji, B., Moore, C. L., Zetsche, B., Volz, S. E., Zhang, F., Shoulders, M. D., et al. (2017). Multidimensional chemical control of CRISPR–Cas9. *Nature Chemical Biology* 13, 9–11. doi:10.1038/nchembio.2224.

Marino, N. D., Pinilla-Redondo, R., Csörgő, B., and Bondy-Denomy, J. (2020). Anti-CRISPR protein applications: natural brakes for CRISPR-Cas technologies. *Nature Methods* 17, 471–479. doi:10.1038/s41592-020-0771-6.

Mayo-Muñoz, D., He, F., Jørgensen, J. B., Madsen, P. K., Bhoobalan-Chitty, Y., and Peng, X. (2018). Anti-CRISPR-Based and CRISPR-Based Genome Editing of *Sulfolobus islandicus* Rod-Shaped Virus 2. *Viruses* 10, 695. doi:10.3390/v10120695.

Nayeemul Bari, S. M., and Hatoum-Aslan, A. (2019). “Chapter Seventeen - CRISPR–Cas10 assisted editing of virulent staphylococcal phages,” in *Methods in Enzymology*, ed. S. Bailey (Academic Press), 385–409. doi:10.1016/bs.mie.2018.10.023.

Nelles, D. A., Fang, M. Y., O’Connell, M. R., Xu, J. L., Markmiller, S. J., Doudna, J. A., et al. (2016). Programmable RNA Tracking in Live Cells with CRISPR/Cas9. *Cell* 165, 488–496. doi:10.1016/j.cell.2016.02.054.

Nihongaki, Y., Kawano, F., Nakajima, T., and Sato, M. (2015). Photoactivatable CRISPR-Cas9 for optogenetic genome editing. *Nature Biotechnology* 33, 755–760. doi:10.1038/nbt.3245.

Perez-Pinera, P., Kocak, D. D., Vockley, C. M., Adler, A. F., Kabadi, A. M., Polstein, L. R., et al. (2013). RNA-guided gene activation by CRISPR-Cas9-based transcription factors. *Nat Methods* 10, 973–976. doi:10.1038/nmeth.2600.

Polstein, L. R., and Gersbach, C. A. (2015). A light-inducible CRISPR-Cas9 system for control of endogenous gene activation. *Nature Chemical Biology* 11, 198–200. doi:10.1038/nchembio.1753.

Qi, L. S., Larson, M. H., Gilbert, L. A., Doudna, J. A., Weissman, J. S., Arkin, A. P., et al. (2013). Repurposing CRISPR as an RNA-guided platform for sequence-specific control of gene expression. *Cell* 152, 1173–1183. doi:10.1016/j.cell.2013.02.022.

Rath, D., Amlinger, L., Hoekzema, M., Devulapally, P. R., and Lundgren, M. (2014). Efficient programmable gene silencing by Cascade. *Nucleic Acids Research* 43, 237–246. doi:10.1093/nar/gku1257.

Rousseau, B. A., Hou, Z., Gramelspacher, M. J., and Zhang, Y. (2018). Programmable RNA Cleavage and Recognition by a Natural CRISPR-Cas9 System from *Neisseria meningitidis.* *Molecular Cell* 69, 906-914.e4. doi:10.1016/j.molcel.2018.01.025.

Schmidt, F., Cherepkova, M. Y., and Platt, R. J. (2018). Transcriptional recording by CRISPR spacer acquisition from RNA. *Nature* 562, 380–385. doi:10.1038/s41586-018-0569-1.

Shipman, S. L., Nivala, J., Macklis, J. D., and Church, G. M. (2017). CRISPR–Cas encoding of a digital movie into the genomes of a population of living bacteria. *Nature* 547, 345–349. doi:10.1038/nature23017.

Slaymaker, I. M., Gao, L., Zetsche, B., Scott, D. A., Yan, W. X., and Zhang, F. (2016). Rationally engineered Cas9 nucleases with improved specificity. *Science* 351, 84. doi:10.1126/science.aad5227.

Strutt, S. C., Torrez, R. M., Kaya, E., Negrete, O. A., and Doudna, J. A. (2018). RNA-dependent RNA targeting by CRISPR-Cas9. *Elife* 7, e32724. doi:10.7554/eLife.32724.

Tang, W., Hu, J. H., and Liu, D. R. (2017). Aptazyme-embedded guide RNAs enable ligand-responsive genome editing and transcriptional activation. *Nature Communications* 8, 15939. doi:10.1038/ncomms15939.

Waldrip, Z. J., Byrum, S. D., Storey, A. J., Gao, J., Byrd, A. K., Mackintosh, S. G., et al. (2014). A CRISPR-based approach for proteomic analysis of a single genomic locus. *Epigenetics* 9, 1207–1211. doi:10.4161/epi.29919.

Wright, A. V., Liu, J.-J., Knott, G. J., Doxzen, K. W., Nogales, E., and Doudna, J. A. (2017). Structures of the CRISPR genome integration complex. *Science*, eaao0679. doi:10.1126/science.aao0679.

Yan, M.-Y., Yan, H.-Q., Ren, G.-X., Zhao, J.-P., Guo, X.-P., and Sun, Y.-C. (2017). CRISPR-Cas12a-Assisted Recombineering in Bacteria. *Appl. Environ. Microbiol.* 83, e00947-17. doi:10.1128/AEM.00947-17.

Young, J. K., Gasior, S. L., Jones, S., Wang, L., Navarro, P., Vickroy, B., et al. (2019). The repurposing of type I-E CRISPR-Cascade for gene activation in plants. *Communications Biology* 2, 383. doi:10.1038/s42003-019-0637-6.

Yu, Y., Wu, X., Guan, N., Shao, J., Li, H., Chen, Y., et al. (2020). Engineering a far-red light–activated split-Cas9 system for remote-controlled genome editing of internal organs and tumors. *Sci Adv* 6, eabb1777. doi:10.1126/sciadv.abb1777.

Zebec, Z., Manica, A., Zhang, J., White, M. F., and Schleper, C. (2014). CRISPR-mediated targeted mRNA degradation in the archaeon Sulfolobus solfataricus. *Nucleic Acids Research* 42, 5280–5288. doi:10.1093/nar/gku161.

Zetsche, B., Volz, S. E., and Zhang, F. (2015). A split-Cas9 architecture for inducible genome editing and transcription modulation. *Nature Biotechnology* 33, 139–142. doi:10.1038/nbt.3149.

Zhang, X., Wang, J., Cheng, Q., Zheng, X., Zhao, G., and Wang, J. (2017). Multiplex gene regulation by CRISPR-ddCpf1. *Cell Discovery* 3, 17018. doi:10.1038/celldisc.2017.18.
